# Advances in Achieving Opioid Analgesia Without Side Effects

**DOI:** 10.3389/fphar.2018.01388

**Published:** 2018-11-29

**Authors:** Halina Machelska, Melih Ö. Celik

**Affiliations:** Department of Experimental Anesthesiology, Charité – Universitätsmedizin Berlin, Corporate Member of Freie Universität Berlin, Humboldt-Universität zu Berlin, and Berlin Institute of Health, Berlin, Germany

**Keywords:** opioid receptor signaling, opioid side effects, addiction, pain, peripheral opioid analgesia, biased agonists, heteromers, endogenous opioid peptides

## Abstract

Opioids are the most effective drugs for the treatment of severe pain, but they also cause addiction and overdose deaths, which have led to a worldwide opioid crisis. Therefore, the development of safer opioids is urgently needed. In this article, we provide a critical overview of emerging opioid-based strategies aimed at effective pain relief and improved side effect profiles. These approaches comprise biased agonism, the targeting of (i) opioid receptors in peripheral inflamed tissue (by reducing agonist access to the brain, the use of nanocarriers, or low pH-sensitive agonists); (ii) heteromers or multiple receptors (by monovalent, bivalent, and multifunctional ligands); (iii) receptor splice variants; and (iv) endogenous opioid peptides (by preventing their degradation or enhancing their production by gene transfer). Substantial advancements are underscored by pharmaceutical development of new opioids such as peripheral κ-receptor agonists, and by treatments augmenting the action of endogenous opioids, which have entered clinical trials. Additionally, there are several promising novel opioids comprehensively examined in preclinical studies, but also strategies such as biased agonism, which might require careful rethinking.

## Introduction

**FIGURE 1:**
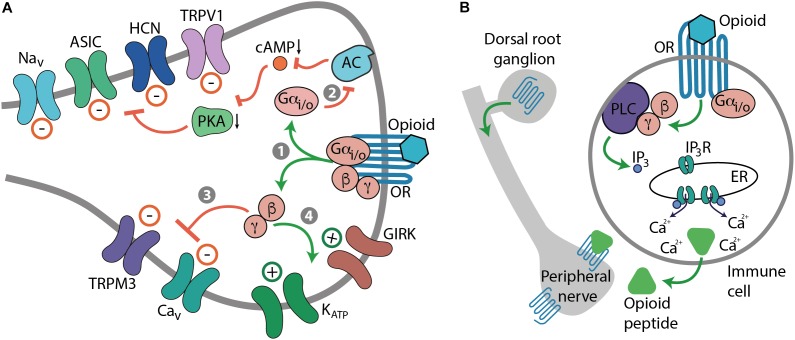
Mechanisms of opioid-induced analgesia. **(A)** Cellular effects mediated by neuronal opioid receptors (OR). Activation of OR by an opioid leads to the dissociation of Gi*/*o proteins into Gαi/o and Gβγ subunits (step 1). Gαi/o inhibits AC, cAMP formation, and PKA activity, which blocks various ion channels, including TRPV1, HCN, ASIC, and Na_v_ channels (path 2). Gβγ blocks Ca_v_ and TRPM3 channels (path 3), and activates GIRK and K_ATP_ channels (path 4). Ultimately, these actions lead to the decrease in neuronal excitability, which culminates in analgesia. **(B)** Cellular effects mediated by OR in immune cells. Activation of leukocyte Gi*/*o-coupled OR leads to the Gβγ-mediated activation of PLC and production of IP_3_, which activates IP_3_R in endoplasmic reticulum (ER) to release intracellular Ca^2+^, which results in the secretion of opioid peptides from immune cells. The released opioid peptides activate neuronal OR and decrease pain.

Opioids relieve pain, but also produce numerous side effects. All actions of opioids are mediated by μ-, δ-, and κ-opioid receptors encoded by the three respective genes (Evans et al., 1992; Kieffer et al., 1992; Mestek et al., 1995; Simonin et al., 1995). Opioid receptors belong to the superfamily of guanine nucleotide-binding protein (G protein)-coupled receptors (GPCRs) and their structures have been solved at high-resolution by X-ray crystallography (Granier et al., 2012; Huang et al., 2015; Che et al., 2018). Upon activation by an agonist, opioid receptors couple to pertussis toxin-sensitive heterotrimeric Gi*/*o proteins, which dissociate into Gαi/o and Gβγ subunits to interact with various intracellular effector systems (Law et al., 2000; Waldhoer et al., 2004; Stein, 2016). Gαi/o inhibits adenylyl cyclases (AC), cyclic adenosine monophosphate (cAMP) formation, and protein kinase A (PKA) activity, which leads to the blockade of a heat sensor transient receptor potential cation channel subfamily V member 1 (TRPV1) (Vetter et al., 2006; Endres-Becker et al., 2007). Gαi/o–cAMP pathway also suppresses hyperpolarization-activated cyclic nucleotide-gated (HCN) channels, acid-sensing ion channels (ASIC), and voltage-gated Na^+^ (Na_v_) channels (Ingram and Williams, 1994; Gold and Levine, 1996; Cai et al., 2014). Gβγ blocks voltage-gated Ca^2+^ (Ca_v_) channels and heat-sensing transient receptor potential cation channel subfamily M member 3 (TRPM3), and activates various K^+^ channels such as G protein-coupled inwardly rectifying K^+^ (GIRK or K_ir_3) channels and adenosine triphosphate-sensitive K^+^ (K_ATP_) channels (Law et al., 2000; Waldhoer et al., 2004; Cunha et al., 2010; Stein, 2016; Dembla et al., 2017). Ultimately, these opioid-mediated actions lead to the suppression of excitatory neurotransmitter release (e.g., substance P, calcitonin gene-related peptide, glutamate), hyperpolarization and an overall decrease in neuronal excitability, which culminates in analgesia (Yaksh, 1997; Ocaña et al., 2004; Stein, 2016; Yudin and Rohacs, 2018) (Figure 1A). Additionally, analgesia can be mediated by opioid receptors expressed in immune cells. Activation of leukocyte opioid receptors leads to the secretion of endogenous opioid peptides (β-endorphin, Met-enkephalin, and dynorphin A 1-17), which involves Gαi/o–Gβγ–phospholipase C (PLC)–inositol 1,4,5-trisphosphate receptor (IP_3_R)– intracellular Ca^2+^ pathway. The released opioid peptides subsequently activate neuronal opioid receptors and alleviate pain (Celik et al., 2016) (Figure 1B).

**FIGURE 2:**
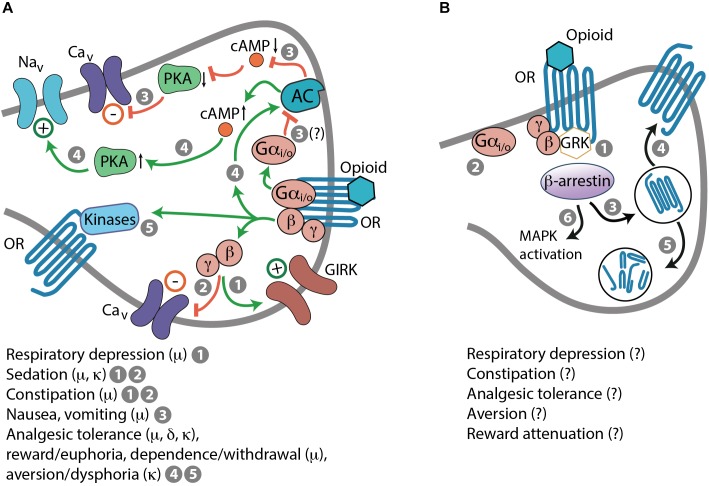
Mechanisms of opioid-induced side effects. **(A)** G protein-mediated side effects in response to activation of opioid receptors (OR). (1) Respiratory depression: Gβγ-dependent activation of GIRK channels. (1 and 2) Sedation and constipation: Gβγ-dependent activation of GIRK channels (1) and inhibition of Ca_v_ channels (2). (3) Nausea and vomiting: Gαi/o-mediated inhibition of AC, decreased cAMP levels and PKA activity, and inhibition of Ca_v_ channels; this is based on indirect evidence (indicated by a question mark). (4 and 5) Analgesic tolerance, reward/euphoria, dependence/withdrawal, or aversion/dysphoria: Gβγ-mediated AC activation, elevated cAMP levels, enhanced PKA activity, and activation of Na_v_ channels (4). Phosphorylation of OR by various kinases (5), including PKA and activated by Gβγ PKC, CaMK II, and MAPK, which results in OR uncoupling form G protein-mediated effects. **(B)** β-arrestin-dependent actions. After even brief activation by an opioid, OR are phosphorylated by GRK recruited by Gβγ, followed by β-arrestin binding to phosphorylated OR (1), which terminates G protein coupling and signaling (2), and leads to OR internalization (3). Dephosphorylated OR can be recycled to the cell membrane (4) or directed to lysosomes and degraded (5). β-arrestin-2 might also promote morphine-induced respiratory depression, constipation, analgesic tolerance, and κ-receptor-mediated aversion, and dampen morphine-induced reward. Some of these effects may involve MAPK activation (6), but mechanisms are unknown (indicated by question marks).

Opioid receptors also mediate numerous adverse effects that limits opioid pain therapy. Activation of μ-receptors can lead to respiratory depression, sedation, constipation, nausea, vomiting, reward/euphoria, and dependence/withdrawal. Activation of δ-receptors can cause convulsions and may produce reward or contribute to rewarding effects of other drugs of abuse. Agonists of κ-receptors exert aversion/dysphoria, sedation, and diuresis (i.e., increased urine output). Each of these symptoms represents a complex phenomenon with multiplex cellular and molecular mechanisms (Kapusta, 1995; Li and van den Pol, 2008; Bruijnzeel, 2009; Koob and Volkow, 2010; Gendron et al., 2016; Dripps et al., 2018). Importantly, these side effects are brought about by G protein-mediated actions in response to opioid receptor activation (Figure 2A). Opioid-induced respiratory depression is mediated by Gβγ-dependent activation of GIRK channels, which results in inhibition of neurons in the brainstem respiratory center (Montandon et al., 2016). Sedation is a consequence of the suppression of neurons in the hypothalamic arousal system, which depends on Gβγ actions on GIRK and Ca_v_ channels (Li and van den Pol, 2008). Constipation results from Gβγ-mediated activation of GIRK channels and inhibition of Ca_v_ channels leading to the suppression of enteric neuronal activity, including acetylcholine and substance P secretion blockade in the gastrointestinal tract (Galligan and Akbarali, 2014). Indirect evidence suggests that nausea and vomiting may involve Gαi/o-mediated decrease of the cAMP–PKA pathway activity, blockade of Ca_v_ channels and thus, inhibition of neurons in the vestibular apparatus (Seseña et al., 2014; Imam et al., 2017). Opioid-induced diuresis results from the inhibition of arginine vasopressin secretion in the hypothalamus, suggestive of G protein involvement, although the exact signaling pathways have not been elucidated (Kapusta, 1995). Chronic opioid use leads to Gβγ–cAMP–PKA pathway activation resulting in enhanced activity of ion channels (e.g., Na_v_ channels) and receptors (e.g., dopamine and *N*-methyl-D-aspartic acid receptors) and thereby, in increased neuronal activity (Nestler and Aghajanian, 1997; Liu and Anand, 2001; Christie, 2008). Furthermore, prolonged activation of opioid receptors results in Gβγ-dependent activation of protein kinase C (PKC), Ca^2+^/calmodulin-dependent protein kinase (CaMK) II, and extracellular signal-regulated kinases 1 and 2 of the mitogen-activated protein kinases (MAPKs). These kinases as well as PKA can phosphorylate opioid receptors, which results in their uncoupling from G protein-mediated effects (Liu and Anand, 2001; Christie, 2008; Al-Hasani and Bruchas, 2011). These events have been ascribed to alterations in opioid receptor signaling underlying analgesic tolerance, reward/euphoria, dependence/withdrawal, or aversion/dysphoria (Nestler and Aghajanian, 1997; Law et al., 2000; Liu and Anand, 2001; Waldhoer et al., 2004; Christie, 2008; Koob and Volkow, 2010; Al-Hasani and Bruchas, 2011; Gendron et al., 2016) (Figure 2A). Additionally, opioid receptors are phosphorylated by GPCR kinases (GRKs), which is followed by recruitment of β-arrestins (Figure 2B). This process occurs after even brief agonist exposure and it terminates G protein coupling and signaling to promote receptor desensitization and internalization. Dephosphorylated opioid receptors can be recycled to the plasma membrane, which reinstates signaling, or can be targeted to lysosomes and degraded (Waldhoer et al., 2004). β-arrestin-2 (also known as arrestin-3) might be involved in morphine-induced analgesic tolerance, respiratory depression, and constipation (Bohn et al., 2000; Raehal et al., 2005). It has also been proposed to mediate κ-receptor-induced aversion via activation of p38 MAPK (Bruchas et al., 2006; Land et al., 2009; Ehrich et al., 2015). Nevertheless, the exact β-arrestin-2-regulated signaling underlying opioid-induced side effects are yet unclear (Figure 2B). The cellular mechanisms of δ-receptor-mediated convulsions have not been identified, and do not seem to involve Gαo or β-arrestin-2 (Dripps et al., 2018).

Clearly, conventional opioids produce numerous side effects, yet they are the strongest painkillers. As all other, non-opioid pain medications also exert adverse actions, none of them produces as powerful pain relief as opioids (Stein and Kopf, 2009; Sondergaard and Gislason, 2017; Welsch et al., 2018). Therefore, opioids will remain the main therapy for moderate and severe pain, which makes efforts to improve their action profile highly desirable and relevant. In the following sections, we present several interesting strategies to achieve safer opioid analgesia, and discuss limitations associated with these new approaches.

## Targeting Opioid Receptors in Painful Tissue

### The Rationale

**FIGURE 3:**
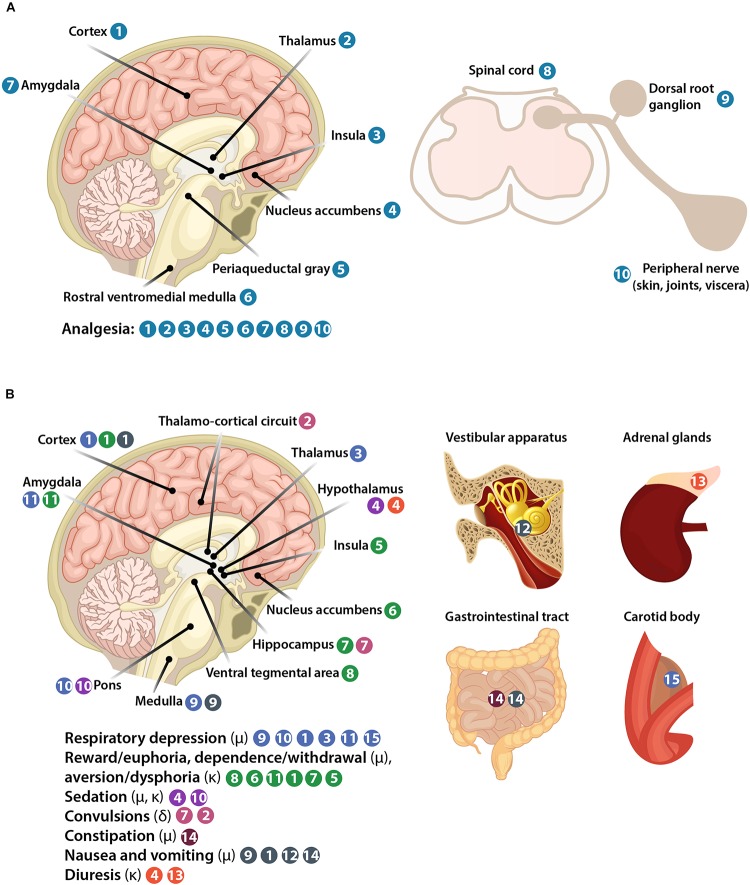
Representation of body structures involved in opioid-induced analgesia **(A)** and side effects **(B)**.

All three opioid receptors (μ, δ, and κ) are expressed in the central nervous system (CNS), including spinal cord and brain, as well as in peripheral sensory neurons (nociceptors). Peripheral opioid receptors are synthetized in nociceptor cell bodies in trigeminal and dorsal root ganglia (DRG), from where they are transported and accumulate in nociceptor peripheral terminals innervating peripheral tissue (skin, joints, viscera) (Figures 1B, 3A). The concept of targeting peripheral opioid receptors comes from the fact that they mediate effective analgesia, but are not involved in fatal effects, in animal models and in humans (Kalso et al., 2002; Stein et al., 2003; Stein and Machelska, 2011; Sawynok and Liu, 2014). Indeed, the serious side effects arise from opioid actions in the brain (Figure 3B). Respiratory depression results from activation of μ-receptors in the brainstem medulla (preBötzinger complex) and pons (Kölliker-Fuse nucleus, parabrachial nuclei, locus coeruleus), in cortical areas, thalamus, and amygdala, and to a lesser extent in the periphery in the carotid body (Pattinson, 2008; Imam et al., 2017). Reward and dependence/withdrawal mediated by μ-receptors, as well as aversion/dysphoria mediated by κ-receptors involve a widely distributed brain network, including the mesolimbic pathway (ventral tegmental area, nucleus accumbens), amygdala, cortex, hippocampus, and insula (Bruijnzeel, 2009; Koob and Volkow, 2010). Sedation is caused by μ- and κ-receptor activation in the hypothalamic and locus coeruleus neurons controlling arousal and sleep (Greco et al., 2008; Li and van den Pol, 2008; Chung et al., 2017). Convulsive actions of δ-receptor agonists involve hippocampus and thalamo-cortical circuits (Jutkiewicz et al., 2006). Constipation is mostly mediated by μ-receptors in peripheral sensory myenteric and submucosal neurons in the gastrointestinal tract, but spinal and supraspinal receptors may also be involved (Burks, 1990; Galligan and Akbarali, 2014; Imam et al., 2017). Nausea and vomiting are mostly mediated by μ-receptors in the medulla, cortex, and vestibular apparatus, and partially in the gastrointestinal tract, possibly secondary to constipation (Porreca and Ossipov, 2009; Imam et al., 2017). Diuresis results from activation of κ-receptors in the hypothalamus with some actions in adrenal glands (Kapusta, 1995). Thus, peripherally restricted opioids should be devoid of the CNS side effects, and produce fewer or less severe adverse actions having both CNS and peripheral components such as constipation, nausea, vomiting (μ-opioids), and diuresis (κ-opioids). Some authors suggested that peripheral μ-receptors mediate morphine-induced analgesic tolerance and paradoxical hyperalgesia, but not analgesia itself, using mice with μ-receptor deletion in TRPV1-expressing neurons (Corder et al., 2017). This is in contrast to studies in mice with μ-receptor deletion in Na_v_1.8-expressing neurons, which showed that peripheral μ-receptors do not contribute to analgesic tolerance or hyperalgesia induced by morphine or its metabolite (Weibel et al., 2013; Roeckel et al., 2017). Further work will be required to find out whether these contradictory findings are related to different μ-receptor-expressing neuronal populations or unidentified knockout strategy-related alterations. Nevertheless, in agreement with the latter studies, experiments without genetic modifications showed that development of tolerance at peripheral μ-receptors is reduced in inflamed tissue in animals and humans, due to the continuous presence of immune cell-derived opioid peptides and enhanced μ-receptor recycling (Stein et al., 1996; Zöllner et al., 2008).

Additional advantage of peripheral opioid receptor targeting is the inhibition of pain at its source, since many painful syndromes originate in peripheral tissue and are usually associated with inflammation (including surgery, arthritis, neuropathy, cancer, and visceral disorders). Under such conditions, opioid receptor synthesis, transport, and G protein coupling in peripheral sensory neurons is increased, and disruption of the perineurial barrier facilitates the access of opioids to receptors (Hassan et al., 1993; Antonijevic et al., 1995; Zöllner et al., 2003; Hackel et al., 2012; Mousa et al., 2007, 2017). Moreover, damaged tissue is infiltrated by immune cells containing opioid peptides and expressing functional opioid receptors (Stein et al., 1990, 1993, 1996; Rittner et al., 2001; Labuz et al., 2009; Boué et al., 2014; Celik et al., 2016). All these events lead to enhanced analgesic efficacy of opioids at peripheral receptors. This has been shown following local application of small, systemically inactive doses of opioids in animal models and in humans (Kalso et al., 2002; Stein et al., 2003; Zeng et al., 2013; Stein, 2016). Importantly, pharmacologic, genetic, and clinical studies have demonstrated that peripheral opioid receptors mediate a large proportion of the analgesic effects produced by systemically administered opioids (Gavériaux-Ruff, 2013; Jagla et al., 2014; Stein and Jagla, 2014; Stein, 2016).

### Reducing Opioid Access to the CNS

**FIGURE 4:**
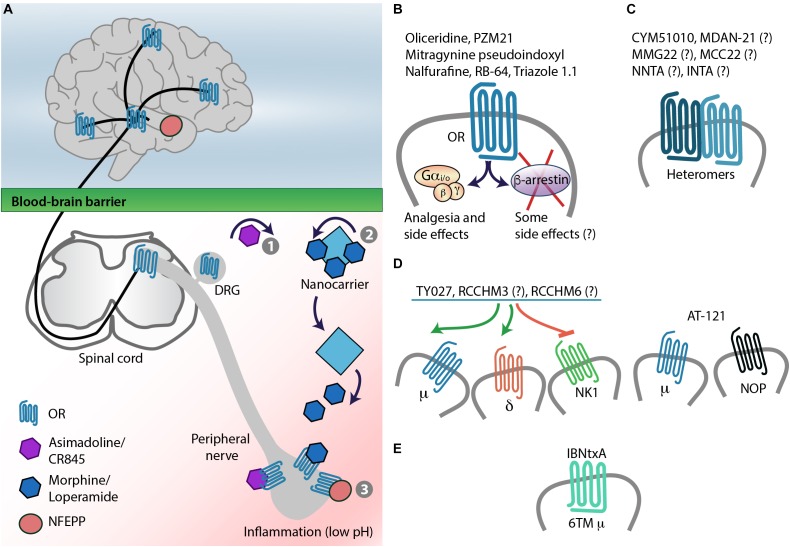
Strategies for safer pain control – targeting opioid receptors. **(A)** Targeting opioid receptors (OR) in peripheral painful tissue by chemical modification of agonists, which results in their decreased blood-brain barrier penetration (1), nanocarrier-based opioid delivery to inflamed tissue (2), or by low pH-dependent OR activation (3). **(B)** Biased agonism: This approach aims at targeting OR–G protein signaling without activation of β-arrestins, which were considered to mediate opioid-induced side effects, but not analgesia. This might need reconsideration, since G proteins not only mediate analgesia but also side effects (see also Figure 2A). **(C)** Targeting heteromers. **(D)** Development of multifunctional ligands, which act as μ- and δ-opioid receptor agonists and NK1 receptor antagonists, or μ- and NOP-receptor agonists. **(E)** Targeting truncated, 6TM domain μ-receptor variants. Question marks indicate that heteromer/multiple receptor selectivity of the ligand was not tested or not confirmed (see also Tables 2, 3).

The above described findings stimulated the development of peripherally restricted opioid receptor agonists by limiting their ability to cross the blood-brain barrier (BBB) (Figure 4A). These strategies focused on κ-opioids, supported by a recent study (Snyder et al., 2018), and include agonist chemical modifications (e.g., incorporation of quaternary structures or amphiphilic molecules which contain hydrophilic and hydrophobic components), and synthesis of peptide-based compounds. However, these modifications often decreased agonist affinity to receptors, which required the use of relatively high doses and did not warrant complete BBB impermeability (Barber and Gottschlich, 1997; Rivière, 2004; Stein and Machelska, 2011). This also applies to peptides, as in contrast to previous beliefs, peptides can cross the BBB (Kastin and Pan, 2010). Nevertheless, two κ-receptor agonists gained pharmaceutical interests, asimadoline (initially termed EMD 61753) and CR845 (formerly FE 202845) (Figure 4A and Table 1). Asimadoline belongs to the amphiphilic molecules and possesses somewhat puzzling action profile. In animal models of hind paw inflammation or sciatic nerve injury, it alleviated pain (Barber et al., 1994) or produced bi-phasic effects, with analgesia at lower doses or shortly after injection, but paradoxically increased pain at higher doses or at later time points (Machelska et al., 1999; Walker et al., 1999). As the analgesic actions were mediated by peripheral κ-receptors, the hyperalgesic effects were either κ-receptor-selective (Walker et al., 1999) or independent of κ- and *N*-methyl-D-aspartic acid receptors (Machelska et al., 1999). Asimadoline was also hyperalgesic in experimental colonic distension model in healthy human volunteers (Delgado-Aros et al., 2003) and tended to enhance postoperative pain in patients undergoing arthroscopic knee surgery (Machelska et al., 1999). In contrast, in preclinical models of visceral inflammatory pain (Gebhart et al., 2000), barostat-induced colonic distension in patients with irritable bowel syndrome (IBS) (Delvaux et al., 2004), and in phase 2b IBS trial (Mangel et al., 2008; Mangel and Williams, 2010), asimadoline was reported to decrease pain. It produced some side effects, which could be of CNS (sedation, headache, dizziness) or both CNS and peripheral origin (diuresis), albeit at higher than analgesic doses (Mangel and Hicks, 2012). These results led to the conclusion that in contrast to somatic pain, asimadoline may be efficacious in the visceral pain, and it is now developed for management of diarrhea-predominant IBS with moderate-to-severe pain (Mangel and Hicks, 2012; Foxx-Orenstein, 2016).

**Table 1:** Novel opioid treatments in clinical trials.

Category	Name/Target	Clinical conditions	Effects	Reference
Agonists with reduced CNS access	Asimadoline^∗^ (peripheral κ-receptors)	Postoperative pain (knee surgery); randomized, double-blind, placebo-controlled; oral	- Tendency to hyperalgesia - No serious side effects (data not shown)	a
		Healthy volunteers (barostat-induced colonic distension); randomized, double-blind, placebo-controlled; oral	- Hyperalgesia - Side effects comparable to placebo (dizziness, nausea, headache)	b
		IBS (barostat-induced colonic distension); randomized, double-blind, placebo-controlled; oral	- Analgesia - Side effects not reported	c
		IBS; randomized, double-blind, placebo-controlled; oral	- Analgesia in D-IBS - No drug-related side effects in analgesic doses^∗^	d
	CR845^#^(peripheral κ-receptors)	Postoperative pain (hysterectomy, bunionectomy); oral, i.v.	- Analgesia - Side effects: dizziness, headache, diuresis	e

Biased agonists	Oliceridine (TRV130) (μ-receptors)	Healthy volunteers (cold pain test); randomized, double-blind, placebo-controlled; i.v.	- Analgesia (superior to morphine) - Side effects: vs. morphine, lesser nausea, similar respiratory depression	f
		Postoperative pain (bunionectomy); randomized, double-blind, placebo-controlled; i.v.	- Analgesia (superior to morphine) - Side effects: constipation, nausea, vomiting, dizziness similar to morphine	g
		Postoperative pain (abdominoplasty); randomized, double-blind, placebo-controlled; i.v. PCA	- Analgesia (similar to morphine) - Side effects: lesser nausea and vomiting vs. morphine	h
	Nalfurafine (κ-receptors)	Approved for uremic pruritus in Japan, but not in Europe	Sedation in analgesic doses (not recommended for pain treatment)	i

DENK inhibitors	PL37, PL265 (enkephalin peptidases)	Postoperative pain (PL37), neuropathic and ocular pain (PL265)	Data not available	j, k

Gene therapy	HSV-PENK (enkephalin overexpression in DRG neurons)	Intractable cancer pain; not randomized, not blinded, not placebo-controlled; intradermal	- Analgesia vs. pre-injection - Side effects: transient and mild injection site erythema and pruritus, body temperature elevation	l
		Intractable cancer pain; randomized, double-blind, placebo-controlled; intradermal	Data not available	m

Agonists with low rate CNS entry	NKTR-181 (μ-receptors)	Osteoarthritis and low back pain; randomized, double-blind, placebo-controlled; oral	Data not available	n


*D-IBS, diarrhea-predominant irritable bowel syndrome; I.v., intravenous; PCA, patient-controlled analgesia. ^∗^Currently under development for D-IBS with moderate-to-severe pain. Sedation, headache, dizziness, diuresis – in higher than D-IBS analgesic dose (Mangel et al., 2008; Mangel and Williams, 2010; Foxx-Orenstein, 2016). ^#^Currently under development for postoperative and osteoarthritis pain. Data in abstracts, press releases, ClinicalTrials.gov; No published peer-reviewed trials. (a) Machelska et al., 1999; (b) Delgado-Aros et al., 2003; (c) Delvaux et al., 2004; (d) Mangel et al., 2008; (e) Albert-Vartanian et al., 2016; (f) Soergel et al., 2014; (g) Viscusi et al., 2016; (h) Singla et al., 2017; (i) Inui, 2012; (j) Roques et al., 2012; (k) http://www.pharmaleads.com; (l) Fink et al., 2011; (m) ClinicalTrials.gov NCT01291901; (n) ClinicalTrials.gov NCT02367820 and NCT02362672.*

**Table 2:** Novel opioid treatments in preclinical models of pathological pain.

Category	Name/Target	Experimental conditions	Effects	Reference
Nanocarrier agonist delivery	anti-ICAM-1 conjugated liposomes loaded with loperamide (μ-receptors in peripheral inflamed tissue)	- CFA hind paw inflammation - Paw pressure test - I.v. or gel on inflamed paw - Blinding (+), R (+), SSE (-)	- Analgesia - Decreased paw volume - Side effects not evaluated	a, b
		- CFA polyarthritis - Paw pressure test - Gel on inflamed paws - Blinding (+), R (+), SSE (-)	- Analgesia - Exacerbated arthritis: higher paw volume, pannus, angiogenesis	c
	PG-morphine (μ-receptors in peripheral inflamed tissue)	- CFA hind paw inflammation - Paw pressure test - Into inflamed paw, i.v. - Blinding (+), R (+), SSE (-)	- Analgesia - No sedation, constipation; 2-fold higher than analgesic doses	d

pH-sensitive receptor activation	**NFEPP** (μ-receptors in peripheral inflamed tissue)	- CFA hind paw inflammation - Hind paw incision - CCI neuropathy - Paw pressure, von Frey, Hargreaves tests - Into inflamed paw, i.v., s.c. - Blinding (+), R (-), SSE (+)	- Analgesia - No sedation, constipation, motor impairment, reward (CPP), respiratory depression (naïve rats); 10-fold higher than analgesic doses	e, f
	FF3 (μ-receptors in peripheral inflamed tissue)	- CFA hind paw inflammation - Hind paw incision - CCI neuropathy - Paw pressure, von Frey, Hargreaves tests - I.v., s.c. - Blinding (+), R (-), SSE (+)	- Analgesia - Sedation, constipation, motor impairment, reward (CPP), respiratory depression (naïve rats); 2.5–10-fold higher than analgesic doses	g

Heteromer bivalent ligands	MMG22 μ-agonist–mGluR5-antagonist (putative μ–mGluR5)	- Lipopolysaccharide (LPS) systemic inflammation - CFA hind paw inflammation - Bone cancer - SNI neuropathy - Tail-flick, von Frey tests - Supraspinal, spinal - Blinding (-,+)^∗^, R (-), SSE (-)	- Analgesia; μ–mGluR5 selectivity not confirmed - No analgesic tolerance, no respiratory depression after spinal injection (LPS or naïve mice); lower than analgesic doses	h, i
	MCC22 μ-agonist–CCR5-antagonist (putative μ–CCR5)	- Sickle disease - von Frey test - Intraperitoneal - Blinding (+), R (+), SSE (-)	- Analgesia; μ–CCR5 selectivity not tested - No analgesic tolerance	j

Multifunctional ligands (μ- and δ-agonists and NK1 receptor antagonists)	**TY027** (CNS μ-, δ-, and NK1 receptors)	- SNL neuropathy - Paw pressure, von Frey, Hargreaves tests - Supraspinal, spinal, i.v. - Blinding (only ferrets), R (only ferrets), SSE (-)	- Analgesia - No constipation, reward (CPP), analgesic tolerance, withdrawal (teeth chattering, wet-dog shakes, diarrhea, weight loss) (naïve rats), vomiting (naïve ferrets); up to 5-fold lower than analgesic doses	k
	RCCHM3, RCCHM6 (CNS μ-, δ-, and NK1 receptors)	- CCI neuropathy - von Frey, cold plate tests - Spinal - Blinding (-), R (-), SSE (-)	- Analgesia - Side effects not evaluated	l

μ-Receptor splice variant agonists	**IBNtxA** (CNS 6TM μ-receptors)	- CFA hind paw inflammation - Zymosan ankle inflammation - SNI neuropathy - von Frey test, facial grimacing; S.c. - Blinding (-,+)^∗^, R (+), SSE (-)	- Analgesia - Less constipation, no reward (CPP), respiratory depression, withdrawal (jumping); analgesic or 2.5-fold higher doses	m, n

Gene therapy	HSV-μ-receptors (overexpressed μ-receptors in DRG neurons)	- SNL neuropathy - von Frey, Hargreaves tests - Into ipsilateral paw - Blinding (+), R (+), SSE (-)	- Reduced basal von Frey sensitivity - Enhanced morphine - and loperamide-analgesia - Side effects not evaluated	o

Endomorphin-1 analog	**Analog 4** (ZH853) (CNS μ-receptors)	- CFA hind paw inflammation - Hind paw incision - SNI neuropathy - Paw pressure, von Frey, Hargreaves tests - Oral, spinal, i.v., s.c. - Blinding (+), R (-,+)^∗^, SSE (-)	- Analgesia - Less analgesic tolerance, motor impairment, reward (CPP, SA), respiratory depression (naïve mice or rats); 2-fold lower or analgesic doses	p, q


*CCI, chronic constriction injury; CFA, complete Freund’s adjuvant; I.v., intravenous; R, randomization; SA, self-administration; S.c., subcutaneous; SNI, spared nerve injury; SNL, spinal nerve ligation; SSE, sample size estimation; TM, transmembrane domain. ^∗^Each sign refers to the corresponding reference (in the citation order). Marked in bold are compounds currently the most comprehensively examined (pathological pain models, various side effects) and showing promising results. (a) Hua and Cabot, 2013; (b) Iwaszkiewicz and Hua, 2014; (c) Hua et al., 2017; (d) González-Rodríguez et al., 2017; (e) Spahn et al., 2017; (f) Rodriguez-Gaztelumendi et al., 2018; (g) Spahn et al., 2018; (h) Akgün et al., 2013; (i) Peterson et al., 2017; (j) Cataldo et al., 2018; (k) Largent-Milnes et al., 2013; (l) Starnowska et al., 2017; (m) Majumdar et al., 2011; (n) Wieskopf et al., 2014; (o) Klein et al., 2018; (p) Zadina et al., 2016; (q) Feehan et al., 2017.*

**Table 3:** Novel opioids tested in animals without pathological pain.

Category	Name/Receptor selectivity	Experimental conditions^∗^	Effects^#^	Reference
Biased ligands	Oliceridine (TRV130) (μ-agonist)	- Tail-flick; S.c. - Blinding (-), R (+), SSE (-)	- Analgesia - No analgesic tolerance - Robust constipation, reward (ICSS)	a
	PZM21 (μ-agonist; also κ-antagonist *in vitro*)	- Tail-flick, hot plate - Hind paw inflammation (30 min); S.c. - Blinding (only hot plate), R (-), SSE (-)	- Analgesia (not in tail-flick) - Less constipation, no respiratory depression, reward (CPP)	b
		- Hot plate; S.c., i.p. - Blinding (+), R (+), SSE (+)	- Analgesia - Respiratory depression - Tolerance to analgesia, but not to respiratory depression; side effects in analgesic or 2-fold higher doses	c
	Mitragynine pseudoindoxyl (μ-agonist; also δ-, κ-antagonist *in vitro*)	- Tail-flick - S.c., oral, supraspinal - Blinding (-), R (-), SSE (-)	- Analgesia - Less constipation, withdrawal (jumping), respiratory depression, no reward, aversion (CPP/CPA)	d
	RB-64 (κ-agonist)	- Hot plate; S.c. - Blinding (-), R (-), SSE (-)	- Analgesia - No sedation, motor impairment, aversion/anhedonia in ICSS - Robust aversion in CPA	e
	Triazole 1.1 (κ-agonist)	- Tail-flick; S.c., i.p. - Blinding (-), R (-), SSE (+)	- Analgesia - No sedation, aversion (ICSS)	f

Heteromer ligands	CYM51010 (μ–δ heteromer agonist)	- Tail-flick - S.c., i.p., spinal - Blinding (-), R (-), SSE (-)	- Analgesia (partially reversed by μ–δ-specific antibody) - Less analgesic tolerance, diarrhea, body weight loss; No change in jumping, teeth chattering, tremor	g
	MDAN-21 bivalent μ-agonist–δ-antagonist (μ–δ heteromers)	- Tail-flick - I.v., supraspinal - Blinding (-), R (-), SSE (-)	- Analgesia (μ–δ selectivity not tested) - No analgesic tolerance, withdrawal (jumping), reward (CPP)	h,i
	NNTA (monovalent agonist of putative μ–κ heteromers)	- Tail-flick - I.v., supraspinal, spinal - Blinding (-), R (-), SSE (-)	- Analgesia (μ–κ selectivity not tested) - No analgesic tolerance, withdrawal (jumping), reward (CPP) - Strong aversion (CPA)	j
	INTA (monovalent agonist of putative μ–κ and/or δ–κ heteromers)	- Tail-flick - S.c., supraspinal, spinal - Blinding (-), R (-), SSE (-)	- Analgesia (μ–κ or δ–κ selectivity not tested) - No analgesic tolerance, aversion (CPA) - Strong reward (CPP)	k

Multifunctional ligands	AT-121 (μ- and NOP-agonist)	- Rhesus monkeys - Naïve and capsaicin - Tail immersion - S.c. - Blinding (+), R (-), SSE (-)	- Analgesia (μ- and NOP-selective) - No analgesic tolerance, scratching, reward (SA), respiratory depression, withdrawal (increased respiration, heart rate, arterial pressure); up to 10-fold higher than analgesic doses	l

Ligands with low rate CNS entry	NKTR-181 (μ-agonist)	- Hot plate - Writhing test - Oral - Blinding, R (+; but not for SA and rigidity), SSE (-)	- Analgesia; receptor selectivity and action site not tested - No reward (SA), mild muscle rigidity and motor impairment at the most effective analgesic doses	m


*I.p., intraperitoneal; I.v., intravenous; R, randomization; SA, self-administration; S.c., subcutaneous; SSE, sample size estimation. ^∗^Experiments were performed in mice or rats, unless otherwise stated. ^#^Side effects were tested in analgesic or lower doses, unless otherwise stated. (a) Altarifi et al., 2017; (b) Manglik et al., 2016; (c) Hill et al., 2018; (d) Váradi et al., 2016; (e) White et al., 2015; (f) Brust et al., 2016; (g) Gomes et al., 2013; (h) Daniels et al., 2005; (i) Lenard et al., 2007; (j) Yekkirala et al., 2011; (k) Le Naour et al., 2014; (l) Ding et al., 2018; (m) Miyazaki et al., 2017.*

CR845 is a tetrapeptide currently under development by Cara Therapeutics (Stamford, CT, United States) for postoperative and osteoarthritis pain. Its analgesic effects were reported in animal models of pancreatitis, abdominal, inflammatory, and neuropathic pain. Completed phase 2 clinical trials stated that CR845 attenuated postoperative pain after laparoscopic hysterectomy and in some patients after bunionectomy, and it was well tolerated with repetitive dosing over 2 weeks in patients with osteoarthritis of knee or hip. The side effects were considered mild and, similar to asimadoline, they included dizziness, headache, and diuresis. However, these data were only presented in abstracts, press releases, and at the ClinicalTrials.gov website (Albert-Vartanian et al., 2016), and thus, independent, peer-reviewed studies will be essential to verify these findings.

### Nanocarrier-Based Approaches

A promising strategy to alter the pharmacokinetic profile and improve therapeutic effects of drugs is the use of nanoparticles as drug carriers. Nanoparticles are defined as molecules of 1–100 nm in at least one dimension, and examples include liposomes, micelles, and polymer-based particles. They have been widely examined for tumor-directed delivery of chemotherapeutics to reduce their off-target toxicity (Cheng et al., 2012). Similar strategies have recently been used to deliver opioids to peripheral inflamed tissue (Figure 4A). Liposomes conjugated with an antibody to intercellular adhesion molecule-1 (anti-ICAM-1) were employed to mimic the properties of immune cells (Hua and Cabot, 2013). Indeed, it has earlier been shown that similar to selectins and integrins α4 and β2 (Machelska et al., 1998, 2004), ICAM-1 expressed on vascular endothelium mediates the migration of opioid peptide-containing immune cells to peripheral inflamed tissue to locally alleviate pain (Machelska et al., 2002). Accordingly, intravenously injected anti-ICAM-1-conjugated liposomes loaded with μ-receptor agonist loperamide accumulated in inflamed tissue and alleviated mechanical hypersensitivity via local opioid receptors in a rat model of unilateral hind paw inflammation (Hua and Cabot, 2013). In the same model, analgesic effects were exerted by loperamide-encapsulated liposomal gel applied topically on the inflamed paw (Iwaszkiewicz and Hua, 2014). In both cases, anti-inflammatory effects were also observed, and all actions of loperamide-loaded liposomes were superior to either conventional loperamide or loperamide gel, respectively. However, in the rat model of polyarthritis, despite producing analgesia, loperamide liposomal gel unexpectedly exacerbated arthritis (Table 2). As the opioid receptor-selectivity has not been tested, the mechanisms of these actions are currently unclear (Hua et al., 2017).

Another nanocarrier-based approach utilized hyperbranched, dendritic polyglycerols (PG) to deliver morphine to peripheral inflamed tissue. Morphine was covalently bound to PG via cleavable ester linker sensitive to esterases and low pH (González-Rodríguez et al., 2017). The rationale was that due to its high molecular weight and hydrophilicity, such PG-morphine injected intravenously will not cross the BBB, but will selectively extravasate from leaky blood vessels characteristic of inflamed tissue. The local low pH and leukocyte esterases will then trigger the release of morphine from PG-morphine to ameliorate pain (Fleige et al., 2012; Nehoff et al., 2014). Indeed, in contrast to morphine, intravenous PG-morphine exclusively produced analgesia via peripheral opioid receptors in painful tissue without sedation and constipation, in a rat model of unilateral hind paw inflammation (Table 2). Consistent with these actions, free morphine was only measured in inflamed paw tissue, but not in the contralateral, non-inflamed paw tissue, blood, and brain (González-Rodríguez et al., 2017). Together, although polyglycerols are biocompatible (Kainthan et al., 2006), the organ toxicity and broader side effect profile, including abuse potential and effects on respiration of PG-morphine need to be investigated to strengthen the clinical applicability of this strategy.

### Painful Tissue-Specific Opioid Receptor Activation

Recent studies explored the opioid receptor–ligand interactions that are specific to pathological painful conditions such as acidosis (pH 5–7 vs. 7.4 in non-inflamed tissue) (Spahn et al., 2017). An agonist designed to fulfill such requirements could freely distribute throughout the whole body, including the brain, but would only activate opioid receptors in peripheral inflamed tissue (Del Vecchio et al., 2017) (Figure 4A). This has been achieved by lowering the dissociation constant (p*K*a) of the μ-receptor agonist fentanyl to the acidic pH. Accordingly, fluorination of fentanyl (p*K*a 8.43) by computer simulations resulted in a design of a novel compound NFEPP [(±)-*N*-(3-fluoro-1-phenethylpiperidine-4-yl)-*N*-phenyl propionamide] with a p*K*a 6.8, which can only be protonated, and thus bind to receptors, at lower than physiological pH. Indeed, *in vitro* experiments confirmed that NFEPP bound to and activated μ-receptors only at acidic pH, whereas fentanyl was active at both acidic and physiological pH. Importantly, unlike fentanyl, intravenously applied NFEPP produced analgesia by activation of opioid receptors exclusively in peripheral injured tissue in rat models of unilateral hind paw inflammation or surgical incision (Spahn et al., 2017), sciatic nerve injury-induced neuropathy, and abdominal pain (Rodriguez-Gaztelumendi et al., 2018). Furthermore, NFEPP did not induce respiratory depression, sedation, motor impairment, reward (assessed by conditioned place preference; CPP), and constipation, even at doses 10-fold higher than the most effective analgesic doses (Spahn et al., 2017) (Table 2). As this compound will not be an option for patients with CNS inflammation, it represents a promising analgesic for pain conditions associated with peripheral tissue damage, which needs to be demonstrated in clinical trials.

Interestingly, another fentanyl derivative FF3 ((±)-*N*-[1-(2-fluoro-2-phenylethyl)piperidine-4-yl]-*N*-phenyl propionamide) with a higher than NFEPP’s p*K*a, 7.22 (but still lower than that of fentanyl), produced injury-restricted analgesia in rat models of inflammatory, surgical, neuropathic, and abdominal pain, similarly to NFEPP. However, unlike NFEPP, FF3 induced side effects, including respiratory depression, sedation, motor impairment, reward, and constipation, at 2.5–10-fold higher than analgesic doses (Table 2). These results suggest that a ligand’s p*K*a should be close to the pH of injured tissue to obtain analgesia without side effects (Spahn et al., 2018).

## Biased Agonism

### Background

The concept of biased agonism (or functional selectivity) is based on the ability of different ligands of the same receptor to stabilize various receptor active states, which leads to the activation of diverse signaling pathways – a biased agonist preferentially activates one signaling pathway over another. Some biased agonists of GPCRs, including opioid receptors, might activate G protein-mediated pathway, whereas others might involve β-arrestin-2. The role of β-arrestin-2 was first examined in the μ-receptor function using β-arrestin-2 knockout mice. These studies used naïve mice, without pathological pain, and reported that morphine induced more efficacious and prolonged analgesia in acute heat pain tests, absent (in hot plate test) or delayed (in tail-flick test) analgesic tolerance, and decreased constipation and respiratory depression in β-arrestin-2 knockout compared to wild-type mice (Bohn et al., 1999, 2000, 2002; Raehal et al., 2005). Whereas naloxone-precipitated morphine withdrawal was unchanged (Bohn et al., 2000), morphine-induced hypothermia and reward (CPP) were substantially enhanced in β-arrestin-2 knockout mice (Bohn et al., 1999, 2003) (Figure 2B). Of note, analgesic tolerance and naloxone-precipitated withdrawal following injection of other μ-receptor agonists such as fentanyl, oxycodone, and methadone did not differ between β-arrestin-2 knockout and wild-type mice (Raehal and Bohn, 2011). Intriguingly, opposite effects were observed using GRK3 knockout mice, who showed weaker acute analgesic tolerance to fentanyl, oxycodone, and methadone, but not to morphine (Melief et al., 2010). Further work of that group indicated that analgesic tolerance to fentanyl involves GRK3/arrestin and c-Jun N-terminal kinase-2 belonging to the MAPK family, whereas tolerance to morphine also involves this kinase, but in GRK3/arrestin-independent manner (Kuhar et al., 2015). It is unclear whether these contradictory results relate to different actions mediated by GRK3 and β-arrestin-2 in response to μ-receptor activation, or to other, unknown alterations resulting from knockout strategies, which cannot be excluded, since GRKs and β-arrestins interact with many GPCRs, not only with opioid receptors (Reiter et al., 2012). Regardless of the discrepancies, these findings suggest that GRK3 and β-arrestin-2 are not essential for side effects exerted by μ-agonists, and that β-arrestin-2 might actually be required for dampening the reinforcement/abuse potential of morphine. Nevertheless, the following efforts focused on design of agonists without or with minimal β-arrestin-2 recruitment properties, but with bias toward G protein-mediated signaling (Figure 4B).

It is currently accepted that biased agonism occurs at all three opioid receptors (Al-Hasani and Bruchas, 2011; Siuda et al., 2017). There is *in vitro* evidence that δ-receptors can adopt distinct receptor conformations in response to different agonists, and that agonist-dependent δ-receptor trafficking and different arrestin isoform recruitment may have behavioral implications (Vicente-Sanchez and Pradhan, 2017). However, no biased δ-receptor agonists with a potential distinction between analgesic actions and undesirable effects such as convulsions have been developed so far. Therefore, the following sections focus on μ- and κ-opioid receptors.

### μ-Receptor Biased Ligands

The first G protein-biased μ-receptor agonist was oliceridine (formerly TRV130) (DeWire et al., 2013) and initially it was classified as potent analgesic with reduced side effect profile (Kingwell, 2015). However, closer analysis of the data and subsequent studies appear less consistent. In mice, oliceridine produced similarly effective analgesia in acute heat pain test, but less constipation compared to morphine. Both agonists also exerted comparable analgesia in a short-lasting (24 h) post-operative pain model in rats. Respiratory function was not affected by either opioid at the most effective analgesic doses, but it was to a similar degree diminished by approximately 2.5-fold (oliceridine) or 4-fold (morphine) higher doses in naïve rats (DeWire et al., 2013). Subsequent study in rodents confirmed oliceridine-induced analgesia and lack of analgesic tolerance in acute heat pain test, but also reported robust constipation and abuse-related behavior in intracranial self-stimulation (ICSS) assay (Altarifi et al., 2017) (Table 3). In healthy human volunteers, compared to morphine, oliceridine exerted superior analgesia in experimental cold pain test, less severe nausea, and comparable degree, but shorter-lasting respiratory depression, which paralleled the time-course of its analgesic effect (Soergel et al., 2014). In phase 2 trial examining patients undergoing bunionectomy, oliceridine produced greater post-operative pain relief, but similar to morphine side effects characterized by the percentage of patients experiencing constipation, nausea, vomiting, and dizziness, as well as by the severity and number of these events (Viscusi et al., 2016). The most recent phase 2b study in patients undergoing abdominoplasty reported comparable rescue analgesic use and reduction in pain intensity, but significantly lower percentage of patients experiencing nausea and vomiting following oliceridine vs. morphine treatment. Whereas earlier clinical trials used fixed-dose design, in that latest study opioids were delivered on an as-needed basis via patient-controlled analgesia (Singla et al., 2017) (Table 1). Together, as all so far performed pre-clinical and clinical studies consistently showed analgesia induced by oliceridine, its side effect profile appears more variable across the studies with most reporting comparable to morphine adverse actions. Additional limitation is the abuse liability of oliceridine (Altarifi et al., 2017) and possibly of other G protein-biased μ-receptor agonists (Bohn et al., 2003).

G protein-biased ligands with μ-receptor agonistic activity, but also affinities to other opioid receptors were later described. PZM21 was initially characterized as μ-receptor agonist with κ-receptor antagonistic activity, and no β-arrestin-2 recruitment. It was reported to produce analgesia in acute heat pain test and in short-lasting (30 min) hind paw inflammation in mice, but no respiratory depression and rewarding (CPP) properties, and less constipation than morphine. However, the side effects were examined in equivalent or lower than the most effective analgesic doses (Manglik et al., 2016). Furthermore, in contrast to that report, a recent study re-examining PZM21 found that it induced respiratory depression similarly to morphine. Additionally, following repeated administration, tolerance developed to PZM21-induced analgesia but not to respiratory depression (Hill et al., 2018) (Table 3).

Mitragynine pseudoindoxyl is a derivative of the natural product mitragynine, which *in vitro* preferentially activated G protein without β-arrestin-2 recruitment, and acted as μ-receptor agonist as well as δ- and κ-receptor antagonist. *In vivo* it produced μ-receptor-mediated analgesia in acute heat pain test, delayed analgesic tolerance, lesser constipation, naloxone-precipitated withdrawal and respiratory depression, and no reward compared to morphine or aversion compared to the κ-receptor agonist U50,488H (CPP/conditioned place aversion; CPA). The doses of mitragynine pseudoindoxyl used to examine side effects were higher than ED_50_, but still substantially lower than the most effective analgesic doses (Table 3). Additionally, the relative contribution of its μ-receptor agonist/δ- and κ-receptor antagonist activity and G protein bias to the improved side effect profile is unclear (Váradi et al., 2016).

A recent paper suggested that just the occurrence of biased signaling might be insufficient, and the degree of bias or bias factor (which quantitatively defines the preference for one signaling pathway over another) closer predicts the opioid therapeutic window (i.e., the separation of doses that produce analgesia and doses that produce side effects). Thus, the higher the G protein bias factor the better the therapeutic window, as calculated for several μ-receptor agonists by comparing respiratory depression and analgesia. Generally, the authors found a correlation between the bias factor and therapeutic window. Nevertheless, it is difficult to clearly define the best bias factor, since it strongly depended and substantially varied with the *in vitro* assays and conditions (e.g., cell line, native tissue, mouse vs. human μ-receptors, signaling pathway type). Similarly, the therapeutic window varied with the pain tests (tail-flick or hot plate) and respiratory depression parameters (arterial oxygen saturation or breath rate). For example, for the most G protein-biased compound SR17018, the G protein bias factor varied from 40 to 102 and therapeutic window for respiratory depression vs. analgesia ranged from 26 to 105 (Schmid et al., 2017); the bias factor of 3 was calculated for oliceridine (DeWire et al., 2013). Furthermore, the correlation between the bias factor and therapeutic window in pathological pain models and for other opioid side effects (constipation, reward, physical dependence) is unknown.

### κ-Receptor Biased Ligands

Nalfurafine (previously TRK-820), first synthetized and characterized in the late 1990s, is a κ-receptor agonist with particularly strong G protein bias at human κ-receptors (bias factor of 300 vs. 7 for rat κ-receptors) (Schattauer et al., 2017). It was initially described as efficacious analgesic and antipruritic with favorable side effect profile; however, a recent study demonstrated its aversive/anhedonic effects (in ICSS assay) in rats (Lazenka et al., 2018). Furthermore, it produced severe sedation at analgesic doses in humans, but lower doses decreased pruritus without severe side effects. Nalfurafine is currently used in Japan for the treatment of uremic pruritus in individuals undergoing hemodialysis (Inui, 2012), but was not approved in Europe, and it is not recommended for the treatment of pain (Inui, 2012)^1^ (Table 1).

RB-64 is a derivative of salvinorin A, an active psychotropic ingredient of a plant *Salvia divinorum*, with a G protein/β-arrestin-2 bias factor of 96 (vs. 3 for typical κ-agonist U69593) (White et al., 2015). It produced κ-receptor-selective analgesia in acute heat pain test, but did not induce sedation, motor impairment, and aversion/anhedonia in ICSS assay compared to U69593 and salvinorin A; however, it was aversive in the CPA paradigm (Table 3). Additionally, U69593 and salvinorin A produced similar analgesia, sedation, and aversion in wild-type and β-arrestin-2 knockout mice, and only motor impairment was slightly lesser in the latter. These data suggest that analgesia and most side effects induced by κ-opioids are mediated by G protein-, but not by β-arrestin-2-dependent signaling. Thus, although RB-64 did not recruit β-arrestin-2 *in vitro* (White et al., 2015), it is unclear whether the lack of β-arrestin-2 signaling indeed account for its effects *in vivo*.

Another G protein-biased κ-receptor agonist triazole 1.1 (with G protein/β-arrestin-2 bias factor of 28) produced similar degree analgesia in acute heat pain test and an anti-pruritic activity, but did not decrease dopamine release in the striatum and did not possess sedative and aversive properties (in ICSS test) compared to classic κ-receptor agonist U50,488H (Brust et al., 2016). Still, the fact that triazole 1.1 was not tested in chronic pathological pain models and the analgesic doses from acute pain tests were used to examine side effects pose some limitations (Table 3).

In summary, the idea to separate desirable and undesirable opioid actions by biased agonists stimulated pain research in the last decades. As analgesic effects of μ- and κ-receptor biased agonists were examined in animals without pain or in very short-lasting inflammation (30 min–24 h) (Table 3), it will be essential to use animal models of pathological pain to closer reflect clinical conditions. To broaden therapeutic window, it is desirable to also use doses exceeding the most effective analgesic doses for testing adverse actions, since fatal effects result from overdosing, as in case of respiratory arrest. The potential abuse liability of G protein-biased μ-receptor agonists, as in case of oliceridine, even in the absence of other side effects, must be seriously considered. Clearly, the addictive properties of opioids have led to their misuse and abuse, which resulted in the opioid crisis worldwide (Abdel-Hamid et al., 2016; Novak et al., 2016; Volkow et al., 2018). Although bias factor depends on experimental conditions and cannot be used as an absolute predictor of the ligand action, the degree of bias is often emphasized, but even very high bias factor does not guarantee the absence of side effects, as in case of nalfurafine. Moreover, it was not always clear whether *in vivo* actions of biased agonists indeed resulted from G protein bias and the lack of β-arrestin-2 engagement, or from a complex pharmacological profile or yet unidentified pathways, as in case of mitragynine pseudoindoxyl and RB-64. Considering these issues, including uncertain mechanistic basis for action of biased agonists, it needs to be acknowledged that opioid-mediated side effects do involve G protein-dependent signaling (see Introduction and Figure 2A).

## Heteromers, Bivalent and Multifunctional Ligands

Heteromers are defined as complexes composed of at least two functional receptor units (protomers) and having different biochemical properties than the individual units. Additional criteria include the colocalization and physical interaction of protomers, and the ability to alter heteromer action by heteromer-specific reagents (Gomes et al., 2016). Heteromers might thus potentially exhibit new pharmacology and represent a novel therapeutic target (Figure 4C). *In vitro* studies in heterologous cells indicated heteromerization between opioid receptors to form μ–δ, μ–κ, and δ–κ heteromers, as well as between opioid and other receptors to form heteromers such as μ-opioid–gastrin-releasing peptide receptor, μ-opioid–metabotropic glutamate receptor 5 (mGluR5), μ-opioid–chemokine receptor 5 (CCR5), μ-opioid–neurokinin 1 (NK1) receptor, μ-opioid–cannabinoid 1, and δ-opioid–cannabinoid 1 receptor. There is only scarce evidence that such complexes exist in endogenous systems, and only μ–δ heteromers appear to fulfill the criteria required for heteromerization in native tissue (Gomes et al., 2016). For example, using μ–δ heteromer-specific antibody, this heteromer was detected in cultured DRG neurons and in various pain-related brain areas in mice (Gupta et al., 2010), although some authors question the co-expression of μ- and δ-receptors in DRG neurons (Wang et al., 2018). Screening of a small molecule library identified CYM51010 as the μ–δ heteromer agonist. This compound produced analgesia in acute heat pain test, which was partially reversed by μ–δ heteromer antibody. Compared to morphine, CYM51010 induced lesser analgesic tolerance and less severe diarrhea and body weight loss, but did not improve other signs of naloxone-precipitated withdrawal (jumping, teeth chattering, paw tremor, whole body tremor) (Gomes et al., 2013) (Table 3). A bivalent ligand comprising a μ-receptor agonist (oxymorphone-derived ligand, oxymorphamine) linked to a δ-receptor antagonist (naltrindole) by a 21-atom spacer (MDAN-21) was designed as a putative μ–δ heteromer ligand. This is based on earlier studies reporting attenuation of morphine-induced side effects by blocking δ-receptor function (Gendron et al., 2016). MDAN-21 produced analgesia in the acute heat pain test, but did not induce acute tolerance, naloxone-precipitated jumping, and reward (in CPP assay) compared to morphine in mice; nevertheless, the μ–δ heteromer-selectivity of MDAN-21 action was not shown (Daniels et al., 2005; Lenard et al., 2007) (Table 3).

To target other putative heteromers, several compounds of different chemistry have been generated. Examples of bivalent ligands are MMG22 and MCC22, which exert agonistic action at μ-receptors and antagonistic activity at various receptors mediating pain. The former was designed to target μ–mGluR5 heteromers, as it consists of μ-agonist (oxymorphamine) and mGluR5 antagonist (metoxy-2-methyl-6-(phenylethynyl)-pyridine) connected by a 22-atom spacer. Compared to morphine or the individual pharmacophores, MMG22 was more potent, but similarly efficacious in mouse models of inflammatory, bone cancer (Akgün et al., 2013), and neuropathic pain (Peterson et al., 2017). However, as the latter study showed that MMG22 acted at μ-receptors and mGluR5 as separate monomers rather than heteromers (Peterson et al., 2017), and the examination of side effects was very limited (Akgün et al., 2013), a rigorous evaluation of a broad adverse effect spectrum will be essential to justify the utility of this compound. MCC22 comprises a μ-agonist (oxymorphamine) and CCR5 antagonist (TAK-220) linked by a 22-atom spacer, and was designed to act at μ–CCR5 heteromers. Compared to morphine, MCC22 ameliorated tactile hypersensitivity with similar efficacy, but higher potency and of longer duration, without inducing tolerance, in a mouse model of sickle cell disease. The assessment of receptor specificity and other than tolerance side effects awaits further research (Cataldo et al., 2018) (Table 2).

Monovalent molecules *N*-naphthoyl-β-naltrexamine (NNTA) and *N*-2′-indolylnaltrexamine (INTA) were developed to target heteromers containing κ-receptors, probably because κ-receptor activation does not induce reward. NNTA designed to act at μ–κ heteromers produced analgesia in acute heat pain test, little tolerance, and no naloxone-precipitated jumping. It also did not induce reward at half-maximal analgesic doses, but exerted strong aversion at maximal analgesic doses (in CPP/CPA paradigm), which is consistent with the pharmacology of mixed κ-receptor agonist/μ-receptor antagonist opioid class (Yekkirala et al., 2011). INTA designed to target μ–κ and/or δ–κ heteromers did not induce acute analgesic tolerance in heat pain test, and was not aversive, but produced robust reward (Le Naour et al., 2014). It is still unclear whether NNTA and INTA exert the respective heteromer-selective effects, and their aversive or rewarding properties are clear drawbacks (Table 3).

Multifunctional ligands are designed to interact with two or more receptors, but heteromers are not necessary their primary target. The advantages of such multitarget, single compounds over a co-administration of several, each receptor-selective ligands, include easier pharmacokinetics and the lack of potential drug–drug interactions. A known, clinically used example of such compound is buprenorphine, which is a partial agonist at μ- and nociceptin/orphanin FQ peptide (NOP) receptors, and a weak antagonist at κ- and δ-receptors. Buprenorphine is predominately applied for the treatment of opioid dependency, as it exhibits ceiling effect for respiratory depression, which diminishes the likelihood of respiratory arrest, and has reduced abuse liability (probably due to its partial μ-receptor agonistic activity) and diminished aversive properties (possibly due to its antagonistic κ-receptor activity). It appears that the majority of clinical trials in cancer pain patients were observational, of poor quality, and with a high risk of bias. Similarly, good quality, randomized studies in neuropathic pain patients are needed. In randomized trials of postoperative or osteoarthritis pain, buprenorphine was concluded to produce fewer respiratory complications, but equivalent analgesia to other opioids (Davis et al., 2018), although the reason for the lack of ceiling analgesic effects in contrast to respiratory depression is unclear, and other studies reported high rate of drop-out due to nausea/vomiting (Fishman and Kim, 2018). Together, as buprenorphine is successfully used for opioid maintenance therapy, the evidence for its analgesic superiority over other opioids in clinical setting appears moderate and more good quality comparative studies are needed (Davis et al., 2018).

Examples of new multifunctional ligands tested in preclinical studies are peptides Tyr-D-Ala-Gly-Phe-Met-Pro-Leu-Trp-NH-Bn(CF3)2 (TY027), RCCHM3, and RCCHM6, which exert μ- and δ-opioid receptor agonistic and NK1 receptor antagonistic activity (Figure 4D). In a very comprehensive study, TY027 injected supraspinally, spinally, or intravenously reversed neuropathy-induced heat and tactile hypersensitivity. In contrast to morphine, TY027 did not produce analgesic tolerance, reward (in CPP test), naloxone-precipitated withdrawal (teeth chattering, wet-dog shakes, diarrhea, weight loss), did not inhibit gastrointestinal transit (in mice or rats), and did not cause retching/vomiting (in ferrets), although the doses were up to 5-fold lower than the most effective analgesic doses. Additionally, TY027 was shown to act as opioid receptor agonist and NK1 receptor antagonist *in vivo* (Largent-Milnes et al., 2013). RCCHM3 and RCCHM6 were efficacious in ameliorating neuropathy-induced tactile and cold hypersensitivity in mice, but the receptor selectivity and side effects were not examined (Starnowska et al., 2017) (Table 2).

Additionally, a bifunctional partial agonist at μ- and NOP receptors, AT-121, has been recently developed. The rationale is based on earlier studies reporting synergistic analgesic actions of morphine and NOP receptor agonists, as well as reduced dopamine release and attenuation of rewarding effects of μ-agonists by NOP receptor agonists (Toll et al., 2016). In rhesus monkeys, subcutaneously injected AT-121 did not induce scratching, but produced comparable to morphine analgesia, which was reversed by opioid and NOP receptor antagonists in an acute heat pain test. AT-121 also reversed capsaicin-induced sensitivity measured by the same test. Unlike oxycodone, it lacked reinforcing effects in self-administration paradigm, and partially attenuated reinforcing action of oxycodone. Unlike heroin, AT-121 at 10 times the analgesic doses did not compromise respiratory and cardiovascular function (respiration rate, minute volume, heart rate, mean arterial pressure). These parameters were also unchanged after injection of the antagonists, indicating a lack of antagonist-precipitated withdrawal in AT-121-treated monkeys. Moreover, following repeated administration (twice daily for 4 weeks), in contrast to morphine, AT-121 did not produce analgesic tolerance in the heat pain test (Ding et al., 2018) (Table 3). Although understandably, the numbers of monkeys per group were low and the chronic pain could not be examined, these conditions present some limitations. Additionally, since NOP receptors are very widely distributed throughout the nervous system and peripheral tissues, other potential side effects produced by NOP receptor agonists, including motor disturbance, memory impairment, and gastrointestinal complications, need to be considered (Mogil and Pasternak, 2001; Toll et al., 2016).

Together, of all opioid receptor heteromers described in heterologous systems *in vitro*, the μ–δ heteromer might be present and function *in vivo*. However, more research would be needed to develop selective ligands, test them in pathological pain models and in a broad range of side effect tests to justify the targeting of μ–δ heteromer as improved pain therapy. Of numerous ligands designed to simultaneously act at various receptors, TY027 has been thoroughly examined, showed analgesic efficacy in pathological pain and promising side effect profile.

## μ-Receptor Splice Variants

Alternative splicing is a genetic regulation that takes place during gene expression when particular exons (transcriptional sequences) of a gene are either included or excluded from the final mRNA, which may result in generation of multiple protein isoforms (Black, 2003). Among opioid receptors, the alternative splicing of μ-receptor coding exons has been extensively examined and the generation of multiple splice variants in mice, rats, and humans has been revealed. In addition to classic full-length, seven-transmembrane (7TM) domain μ-receptor variants, there are also exon 1-associated truncated 1TM domain variants and exon 11-associated truncated 6TM domain variants. Depending on the species, two to five 1TM domain and 6TM domain variants have been described (Pasternak and Pan, 2013). Several of these variants have been detected in the mouse brain, spinal cord, and DRG at the mRNA level (Pasternak and Pan, 2013; Wieskopf et al., 2014), and some of them were examined by immunohistochemistry, but difficulties associated with the specificity of antibodies preclude the convincing evidence on their expression at the protein level (Pasternak and Pan, 2013). It has been suggested that 1TM domain variants do not bind ligands, but function as molecular chaperones that facilitate expression of the 7TM domain μ-receptor and thereby enhance morphine analgesia. In contrast, 6TM domain variants appear to possess distinct pharmacology characterized by the use of a compound iodobenzoylnaltrexamide (IBNtxA) (Figure 4E). Radiolabeled IBNtxA-binding sites were detected in the brain membrane homogenates in wild-type mice and mice lacking 7TM domain μ-, δ-, and κ-opioid receptors, but were absent in exon 11 knockout mice (Majumdar et al., 2011). Systemically applied IBNtxA diminished spontaneous inflammatory pain and mechanical hypersensitivity in inflammatory and neuropathic pain models in wild-type mice, whereas the effects in the latter two models were absent in exon 11-lacking mice (Wieskopf et al., 2014). Compared to morphine, IBNtxA at analgesic or higher doses exerted lesser constipation, and did not produce respiratory depression, naloxone-precipitated jumping, and CPP reward (Majumdar et al., 2011) (Table 2). Experiments in μ-receptor knockout mice reconstituted with 6TM domain variants confirmed their contribution to IBNtxA-induced analgesia (in the acute heat pain test) (Lu et al., 2018). It is still unclear what cellular mechanisms underlie analgesic effects and improved side effect profile of 6TM domain variants, and whether these variants are functional in humans. The complexity of this system is additionally implied by animal studies describing excitatory cellular actions of 6TM domain variants and enhanced heat sensitivity following repetitive injections of IBNtxA in naïve mice (Convertino et al., 2015; Samoshkin et al., 2015).

## Targeting Endogenous Opioid Peptides

### Enkephalinase Inhibitors

**FIGURE 5:**
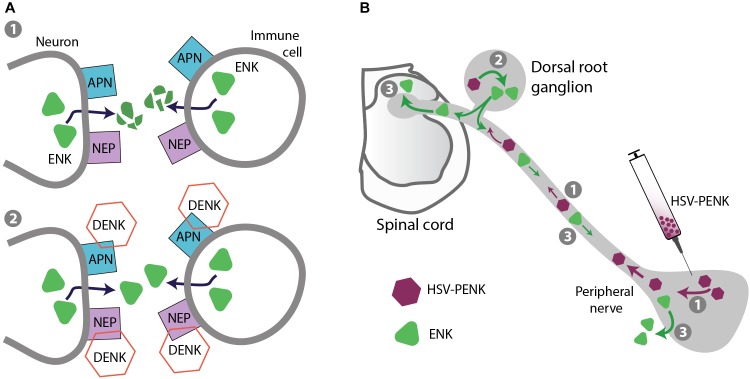
Strategies for safer pain control – targeting endogenous opioid peptides. **(A)** Prevention of opioid peptide degradation. (1) Opioid peptides, including enkephalins (ENK) are degraded by APN and NEP expressed on neurons (central and peripheral) and immune cells in inflamed tissue. (2) DENK inhibitors block APN and NEP, and prevent ENK degradation to locally alleviate pain. **(B)** Gene transfer to enhance opioid peptide production in native tissue. As an example, HSV vector encoding ENK precursor PENK injected into peripheral tissue is taken up by peripheral terminals of dorsal root ganglion (DRG) neurons and transported to their cell bodies in DRG (1), where PENK is processed to ENK (2). ENK is then transported to peripheral and central DRG neuron terminals (3), released, and respectively activates peripheral and spinal opioid receptors to produce analgesia.

Enhancing the activity of endogenous opioid peptides as natural agonists of opioid receptors represents an intrinsic pain control. Opioid peptides, including β-endorphin, enkephalins and dynorphins are expressed in neurons in pain-relevant regions of the central or peripheral nervous system as well as in immune cells accumulating in peripheral painful tissue (Fields, 2004; Stein and Machelska, 2011). Hence, targeting endogenous opioids at the site of their native expression may diminish the risk of off-site, unphysiological actions. Electrical stimulation of periventricular/periaqueductal gray matter and thalamus, or activation of immune cells in peripheral inflamed tissue (by surgery-related stress or local application of opioid peptide-releasing agents) alleviates pathological pain involving endogenous opioids in humans (Stein et al., 1993; Bittar et al., 2005; Likar et al., 2007). Notably, immune cell-derived opioid peptides exerted additive/synergistic analgesic action with peripherally (intra-articularly) applied morphine in patients with postoperative pain (Stein et al., 1996; Likar et al., 2004), which may be related to the activation of leukocyte opioid receptors (Celik et al., 2016). Nonetheless, opioid peptides are rapidly enzymatically degraded, and the best characterized enzymes are aminopeptidase N (APN; also known as CD13) and neutral endopeptidase (NEP; also known as neprilysin, CD10, or enkephalinase). Among opioid peptides, the predominant substrates of APN and NEP are Met- and Leu-enkephalin, but dynorphin A 1-17 can also be inactivated. Both enzymes are functional in the CNS, peripheral nerves, and immune cells, and their blockade prevented opioid peptide degradation (Bourgoin et al., 1986; Le Guen et al., 2003; Schreiter et al., 2012). Since the actions of both peptidases are complementary, their concomitant blockade is most efficient, which led to the development of dual APN and NEP inhibitors, now known as dual enkephalinase (DENK) inhibitors (Figure 5A). Over the last four decades, numerous DENK inhibitors have been synthetized and found to alleviate inflammatory, neuropathic, abdominal, cancer, and postoperative pain, when applied intravenously, orally, or into inflamed tissue in animal models (Roques et al., 2012; Schreiter et al., 2012). Compared to morphine, DENK inhibitors in analgesic or higher doses produced no or less severe side effects, including tolerance, naloxone-precipitated withdrawal, reward (CPP, ICSS), respiratory depression, and constipation (Noble and Roques, 2007). Within the last decade, DENK inhibitors developed by Pharmaleads (Paris, France) for the treatment of postoperative pain (PL37) or neuropathic and ocular pain (PL265) entered clinical trials, but the data are not yet available (Roques et al., 2012)^2^ (Table 1).

### Gene Therapy

Gene therapy (or gene transfer) is based on the introduction of DNA or RNA encoding a protein of interest, and offers a possibility of the protein long-term expression in native tissue. For *in vivo* delivery of genes encoding enkephalin precursor proenkephalin (PENK) or β-endorphin precursor proopiomelanocortin (POMC), different vectors have been used, including plasmids, non-replicating adenoviruses, adeno-associated viruses, and herpes simplex virus (HSV), as well as non-plasmid and non-viral DNA vectors (e.g., MIDGE; minimalistic, immunologically defined gene expression vector). There are numerous preclinical studies, in which these PENK- or POMC-encoding vectors were applied intramuscularly, on the spinal cord, intra-articularly, or into the skin/subcutaneous tissue, which resulted in enhanced expression of the respective peptides (Met/Leu-enkephalin or β-endorphin) in the corresponding tissue. Consequently, these treatments led to attenuation of mechanical and heat hypersensitivity in models of inflammatory, neuropathic, or cancer pain, mediated by spinal or peripheral opioid receptors; these analgesic effects were rather modest, but in some cases persisted for several weeks (Machelska et al., 2009; Simonato et al., 2013; Goss et al., 2014; Hu et al., 2016; Klein et al., 2018). Since this strategy targets peripheral and spinal cord tissue, the opioid side effects mostly mediated in the brain are not anticipated, but this has not been verified. Compared to non-viral vectors, viral vectors have higher transfection efficacy, which is attributed to the natural ability of viruses to infect and express their genes in host cells. However, viral vectors can potentially cause toxicity and inflammation, which depends on treatment conditions (e.g., dosing, route of application), although based on so far available data, HSV vectors inoculated into the skin are predicted to be safe (Wolfe et al., 2009; Simonato et al., 2013; Goss et al., 2014). The first phase 1 clinical trial testing this strategy employed HSV-based vector encoding human PENK injected intradermally (into the pain-corresponding dermatomes) in terminally ill patients with intractable cancer pain. The treatment was well tolerated and no serious adverse events were observed. Over the 4-month follow-up, the treatment-emergent adverse effects (injection site erythema and pruritus, and body temperature elevation) were transient and judged of mild severity. The study was very small (four or fewer patients per group), not blinded and not placebo-controlled, but also reported a dose-related decrease of pain (up to 4 weeks post-treatment) as the secondary outcome (Fink et al., 2011). A phase 2, randomized, double-blind, placebo-controlled, multicenter study testing HSV-encoding PENK in patients with intractable malignant pain has been completed, but the data are not yet released (ClinicaleTrials.gov NCT01291901) (Table 1). Based on the corresponding pre-clinical studies it is anticipated that HSV-encoding PENK is taken up by cutaneous terminals of peripheral sensory neurons and axonally transported to their cell bodies in DRG, where PENK is processed to enkephalins. The enkephalins can be then transported toward peripheral and central DRG neuron terminals, released and respectively activate peripheral and spinal opioid receptors to provide analgesia (Antunes Bras et al., 1998, 2001; Goss et al., 2001; Klein et al., 2018) (Figure 5B). Similar strategy can also be used to enhance expression of opioid receptors. For example, HSV-encoding μ-receptors applied to mouse hind paw elevated μ-receptor-immunoreactivity in epidermal skin fibers, DRG cells, and dorsal horn spinal cord, alleviated basal mechanical hypersensitivity, and enhanced analgesic effects of morphine and peripherally acting loperamide injected systemically in a neuropathic pain model (Table 2). Surprisingly and not clarified yet, combined treatment with HSV-encoding μ-receptors and HSV-encoding PENK was ineffective (Klein et al., 2018).

## Other Approaches

### Abuse-Deterrent Opioid Formulations

Currently clinically used opioids have been modified to obtain abuse-deterrent formulations, and several of such substances have been approved by the Food and Drug Administration (Becker and Fiellin, 2017). The general aim was to make these new formulations difficult to inhale or inject, and to get a high from. This has been attempted by means of physical or chemical barriers to hinder crushing, chewing, or solubilization of pills, as in case of modifications of morphine (MorphaBond ER, Arymo ER), oxycodone (OxyContin, RoxyBond, Xtampza ER), and hydrocodone (Vantrela ER, Hyslinga ER). Alternatively, opioid receptor agonists were combined with antagonists, as in case of Embeda (morphine and naltrexone), Troxyca ER (oxycodone and naltrexone), or Targiniq ER (oxycodone and naloxone) (Becker and Fiellin, 2017; Salwan et al., 2018). However, these strategies have not proved successful in preventing opioid abuse. To overcome the obstacles associated with hindering the misuse of these formulations they were taken at higher doses or replaced with other opioids having a higher abuse liability such as heroin or fentanyl (Cicero and Ellis, 2015; Becker and Fiellin, 2017; Curfman et al., 2018; Salwan et al., 2018).

### Agonists With Low Rate CNS Entry

Nektar Therapeutics (San Francisco, CA, United States) has synthetized and been testing a compound NKTR-181, a μ-receptor agonist with a low rate influx across the BBB, proposing that such substance should have lower abuse potential compared to drugs with rapid CNS entry. The slow CNS entry has been achieved by addition of a polyethylene glycol functional group to morphine-like (morphinan) pharmacophore. NKTR-181 produced analgesia in naïve animals in acute heat pain test and in acetic acid-induced writhing model, but the μ-receptor-selectivity and the action site (central, peripheral) have not been examined. Its side effect profile was improved compared to oxycodone, although at the most effective analgesic doses NKTR-181 induced mild muscle rigidity and motor impairment. Compared to cocaine and oxycodone, it did not produce reward in self-administration paradigm (Miyazaki et al., 2017) (Table 3). In healthy, non-physically dependent recreational opioid users, single oral application of NKTR-181 (in doses used in ongoing phase 3 trials) induced significantly lower drug liking effects (indicative of lower abuse potential) and smaller changes in the pupil diameter (indicative of less robust CNS actions) relative to oxycodone. Still, the effects of the highest NKTR-181 dose used were significantly higher vs. placebo (Webster et al., 2018). The compound is considered to be resistant to physical or chemical tampering, albeit the data were not shown (Miyazaki et al., 2017), and the possibility of taking it at high doses to achieve high CNS levels cannot be excluded, as in case of abuse-deterrent opioids. The company sponsored completed phase 2 trial in patients with osteoarthritis (NCT02367820) and phase 3 trial in patients with chronic low back pain (NCT02362672; both at ClinicalTrials.gov), but the peer reviewed data are not available yet (Table 1).

### Endomorphin Analogs

Endomorphin-1 and endomorphin-2 are additional endogenous opioid peptides, although (in contrast to endorphins, enkephalins, and dynorphins) their precursor has not been identified so far (Zadina et al., 1997). Both endomorphins are highly selective at μ-receptors and exerted analgesia with reduced side effects relative to conventional opioids in some preclinical studies. Due to their poor metabolic stability, numerous endomorphin analogs with potentially improved pharmacological properties have been developed (Gu et al., 2017). Example is a recently characterized cyclized, D-amino acid-containing endomorphin 1 peptide analog termed analog 4 or ZH853 (Tyr-c-[D-Lys-Trp-Phe-Glu]-Gly-NH2). This compound alleviated heat and mechanical hypersensitivity in models of neuropathic, inflammatory, and postoperative pain following spinal or intravenous injections. Relative to morphine, the analog 4-induced analgesia was equally effective but longer-lasting (Feehan et al., 2017). Moreover, in contrast to morphine, analog 4 produced lesser analgesic tolerance, no motor impairment, respiratory depression, and reward (in CPP and self-administration paradigms), and did not induce spinal glia activation (Zadina et al., 2016), although side effects were mostly tested using similar or lower than analgesic doses (Feehan et al., 2017) (Table 2). Whereas the results appear promising, considering that the compound crosses the BBB and activates μ-receptors in the brain (Zadina et al., 2016), it will be important to elucidate the mechanistic basis for its improved side effect profile.

### Allosteric Modulators

Allosteric modulators are ligands that bind the allosteric site of the receptor (i.e., the site that does not bind orthosteric ligands such as endogenous and standard exogenous ligands) and can modulate (positively or negatively) the effect of the orthosteric ligand without eliciting activity on its own. For example, it is anticipated that positive allosteric modulators will enhance the activity of endogenous opioid peptides, maintain their temporal and spatial action, and potentially limit the off-target adverse effects. Although several such compounds have been characterized *in vitro*, their utility *in vivo* is yet to be determined (Remesic et al., 2017).

## Conclusion

Conventional opioids are the most effective painkillers, but they also produce adverse affects. Additionally, their prolonged use leads to addiction, which limits the effectiveness of pain therapy and has resulted in a worldwide opioid epidemic (Abdel-Hamid et al., 2016; Novak et al., 2016; Volkow et al., 2018). Therefore, the search for opioids with improved side effect profile and low abuse liability is undisputed. Several novel treatments targeting peripheral κ-receptors (asimadoline, CR845), endogenous opioid peptides (DENK inhibitors, HSV-PENK), and agonist with a low rate CNS entry (NKTR-181) are under development and are tested in clinical trials, but not all results are available yet (Table 1) and it remains to be seen whether they enter clinical practice. The G protein-biased agonism as a safer pain therapy needs to be verified, since opioid-induced adverse actions are mediated by G proteins (Figure 2A), and there are increasing numbers of studies that report biased agonist-induced constipation, sedation, respiratory depression, and addiction (Inui, 2012; Soergel et al., 2014; Altarifi et al., 2017; Hill et al., 2018) (Tables 1, 3). Encouragingly, there are several new opioids examined in preclinical studies, which are comprehensively characterized in various pathological pain models and methods assessing a wide spectrum of side effects, and show promising results. They include an agonist sensitive to low pH characteristic of painful tissue (NFEPP), ligands targeting multiple receptors (TY027) or μ-receptor splice variants (IBNtxA), and endomorphin-1 analog (analog 4 or ZH853) (Table 2). Nevertheless, several aspects are still open such as mechanistic basis of analgesia and improved side effect profile (IBNtxA, endomorphin-1 analog), the need for replication of the initial findings (NFEPP, TY027), and examination of their clinical efficacy. Although there are no preclinical assays that ideally reflect pain in humans, the pathological pain models involving tissue damage and lasting for days or weeks (Table 2) closer resemble clinical conditions than the tests inducing pain lasting for seconds in naïve animals (Mogil, 2009) (Table 3). Additionally, even though there is an increasing awareness of the importance of the rigorous study design and performance, including blinding, randomization, and sample size estimation (Kilkenny et al., 2010; Berg, 2017), many animal studies still do not adhere to these requirements (Tables 2, 3). It is thus critical that all these aspects are considered when the clinical translation of preclinical studies is judged. Finally, it is crucial to recognize the multifactorial biopsychosocial etiology of chronic pain and that it requires a multidisciplinary management comprising not only pharmacologic, but also psychological, and physiotherapeutic approaches (Scascighini et al., 2008; Stein and Kopf, 2009). Pharmacologic treatment alone is insufficient and will always carry a risk of unwanted behaviors, as seen by the shifting trends in pain management and the addiction landscape toward alternative opioid (e.g., loperamide) and non-opioid (e.g., gabapentin, pregabalin), but also potentially dangerous medications (Throckmorton et al., 2018).

## Author Contributions

HM wrote the manuscript. MÖC prepared the figures. HM and MÖC approved the final version of the manuscript.

## Conflict of Interest Statement

The authors declare that the research was conducted in the absence of any commercial or financial relationships that could be construed as a potential conflict of interest.

## Footnotes

1 https://www.ema.europa.eu/documents/medicine-qa/withdrawal-marketing-authorisation-application-winfuran-nalfurafine_en.pdf

2 http://www.pharmaleads.com/
